# Inquest on a Nurse

**Published:** 1910-08-13

**Authors:** 


### INQUEST ON A NURSE.

An inquest was held last week on the body of a nurse, found dead in bed at a house in Anderson Street, Chelsea. Deceased's brother-in-law said she had been in the Mount Vernon Hospital suffering from tuberculosis. She had been told that it was incurable, and since she took up Christian Science she had refused to have a doctor. Ten days before her death she went to live with a lady who was a Christian Scientist at Anderson Street. A Christian Scientist, who took in paying guests, said deceased came a little more than a week ago as a Christian Science patient to her. She paid only a guinea a week, as the witness knew she was not well off. She used to cough at times and walked with a stick owing to her bad legs. She had only Christian Science treatment. Another witness said she was a Christian Science practitioner, and got her living in that way. She had been a Christian Scientist for five years and an established practitioner for about three years. Deceased had written to her and she had treated deceased at the rate of 3s. a week, and later on for 2s. The Coroner: What was the treatment?—Prayer. Anything else?—No; I cannot explain Christian Science treatment; it would take too long, and you would not be able to understand it unless you understood Christian Science. Witness added that she believed that consumption could be healed by Christian Science. The Coroner: If the doctor says it cannot be healed and you say it can, what is the explanation why it was not healed by Christian Science?—I suppose because I had not sufficient understanding to meet it. A local registered practitioner, who was called after death, said the body was emaciated by phthisis. There was also some dropsy. Both lungs were badly affected by tubercle, the left being practically useless. There was no sign of healing, and death was due to exhaustion consequent on tuberculosis. She could have had relief from the dropsy by "tapping." She could not have lived very long; nothing could have been done to save her life, although proper medical care might have prolonged her life a little. Nothing could have healed the lung. The stoppage of a cough in tuberculosis was often a bad sign. The jury returned a verdict of "Death from natural causes."

